# Genetics of educational attainment and social problem behaviors: Robust estimates of direct genetic effects

**DOI:** 10.21203/rs.3.rs-7328005/v1

**Published:** 2025-08-29

**Authors:** Lyydia Alajääskö, Marina Aguiar-Palma, Abdel Abdellaoui, Titus Galama

**Affiliations:** 1 Vrije Universiteit Amsterdam (VU), The Netherlands; 2 Amsterdam UMC, University of Amsterdam, The Netherlands; 3 University of Southern California, Los Angeles, United States

**Keywords:** Social behavioral traits, cognitive/non-cognitive traits, educational attainment, polygenic indices, within-family trio design, sex

## Abstract

This study examines the genetic underpinnings of (problematic) socioemotional behaviors in children (6–18 years old) by leveraging a within-family trio design using data from the Lifelines cohort (N=3,090–4,510). Socio-emotional development is key to understanding long-term educational, occupational, and mental health outcomes. Yet, their genetic foundations are not fully understood. We estimate direct genetic effects of educational attainment (EA)-related polygenic indices (PGIs), as a whole and decomposed into cognitive (Cog) and non-cognitive (NonCog) components, on a comprehensive set of internalizing and externalizing behaviors assessed via self- and parental-reported Achenbach System of Empirically Based Assessment (ASEBA) measures. Our findings reveal that higher EA-related PGIs, particularly their cognitive component, are robustly associated with lower levels of attention problems, rule-breaking, and somatic complaints. These effects persist after adjusting for parental genetics, supporting a causal interpretation. Some associations—such as those of the NonCog EA PGI with attention problems and rule-breaking, and of the full EA PGI with externalizing behaviors—lose statistical significance when parental genetics are included, suggesting that small environmentally mediated parental influences may play a role. We also find notable sex differences, with stronger associations in girls, especially for internalizing traits, and observe modest (although insignificant) differences between self- and parent-reported outcomes. Our findings contribute novel evidence that cognitive genetics influence behavioral development and highlight the value of family-based genetic designs for uncovering the complex interplay of genes and environment in socio-emotional functioning.

## 1 Introduction

Socioemotional behaviors are shaped by complex interactions of genetic, environmental, and developmental factors. Unlike personality traits, which are considered relatively stable, socioemotional behaviors exhibit dynamic patterns of emotional regulation, impulse control, and social interaction that can vary across contexts. Understanding the role of genetic factors in socioemotional behaviors may provide insight into their etiology and help to refine theory.

A growing body of research has demonstrated the importance of social and emotional functioning behaviors in shaping long-term outcomes, including educational attainment, career success, and social functioning (Belfi and Borghans, 2025). Heckman and colleagues emphasize that perseverance and self-control play a crucial role in labor-market success and economic stability, sometimes exceeding the predictive power of cognitive abilities (Heckman and Kautz, 2012; Heckman et al., 2006; Heckman and Rubinstein, 2001). Furthermore, meta-analytic evidence shows that emotional intelligence is a significant predictor of academic performance (MacCann et al., 2020).

Given the central role of social and emotional behaviors in shaping educational outcomes, genetic predictors of educational attainment should also capture variance in these behaviors. Recent advances in genome-wide association studies (GWAS) provide an opportunity to explore such associations. In this paper, we investigate the role of genetic factors in social behavior, estimating robust associations with educational attainment (EA)-related genetic factors, on a comprehensive set of parental- and self-reported socioemotional behaviors.

Large-measure GWASs on educational attainment (EA) have identified thousands of genome-wide significant single-nucleotide polymorphisms (SNPs) associated with years of schooling, with polygenic indices (PGIs), which aggregate the millions of small genetic effects of SNPs across the genome, explaining up to 16% of the variance in EA (Lee et al., 2018; Okbay et al., 2022). EA-related genetic correlates extend beyond educational achievement, demonstrating predictive power for a range of diverse outcomes, including self-control, depression, and personality traits (Belsky et al., 2016; Smith-Woolley et al., 2019; Okbay et al., 2022). Given the substantial sample size of EA GWASs, and therefore predictive power, and the broad predictive validity of EA-related PGIs beyond educational attainment, we use the EA PGI in our analyses to explore its relevance for (problematic) socioemotional behaviors.

Recent work has demonstrated that EA-related genetic influences are multifaceted, encompassing both cognitive and non-cognitive components. Using GWAS-by-subtraction, Demange et al. (2021) developed two orthogonal polygenic indices (PGIs) that isolate the portion of EA-related genetic variance attributable to performance on cognitive tasks from the residual, non-cognitive component. This decomposition offers a powerful empirical tool for probing how distinct dimensions of EA-related genetic variance—cognitive and non-cognitive^1^—are associated with socioemotional behaviors. While the cognitive components (i.e. those related to performance on standardized tests) of EA-related genetics are well-documented, growing attention has been given to the role of non-cognitive factors, such as motivation, self-regulation, and personality (Demange et al., 2021,0; Malanchini et al., 2024). These are increasingly recognized as critical to understanding the broader associations of EA-related genetics on socioemotional behaviors.

The cognitive / non-cognitive decomposition aligns with what Belsky and Harden (2019) term phenotypic annotation—a framework for bridging the interpretive gap between GWAS findings and psychological or developmental mechanisms. Rather than tracing the biological pathways linking individual genetic variants to outcomes, phenotypic annotation emphasizes mapping polygenic associations onto behavioral, developmental, and social phenotypes across the life course. It advocates for shifting focus from specific genes to whole-genome scores, from narrow target traits to broader nomological networks, and from proximate biology to long-run developmental trajectories.

Our study builds on the phenotypic annotation framework by examining how distinct dimensions of EA-related genetic variance—cognitive and non-cognitive—are associated with internalizing and externalizing socioemotional behaviors, as measured by the Achenbach System of Empirically Based Assessment (ASEBA) (Achenbach, 2001), across different reporter perspectives. Internalizing behaviors—processes within the self—are linked to mental health conditions such as depression and anxiety, and include somatic complaints and social withdrawal. Externalizing behaviors, by contrast, manifest as outward-directed actions such as aggression and rule-breaking, often associated with conduct disorders and substance abuse. By relating EA-related PGIs to these behavioral domains across informants, our approach contributes to the broader goal of phenotypic annotation: unpacking the psychological mechanisms through which polygenic influences on educational attainment operate.

The heritability of ASEBA (problem) behaviors has been widely studied, with twin studies consistently reporting moderate to high estimates ranging from 30% to 90% (Hicks et al., 2004; Kendler and Myers, 2014; Kendler et al., 2016; Nikstat and Riemann, 2020). These estimates vary by age, gender, source of symptom reporting, and methodology. Heritability estimates tend to increase with age, while the contribution of shared environment decreases (Nikstat and Riemann, 2020). Studies of adolescents and young adults consistently show higher heritability of externalizing behaviors (Kendler and Myers, 2014; Kendler et al., 2016; Hicks et al., 2004), whereas research in younger children suggests a greater contribution from family-level factors early in development (Franić et al., 2014; de Zeeuw et al., 2020).

Sex differences also shape these patterns. Kendler et al. (2016) observe that genetic factors play an important role in females, whereas shared environmental influences are more prominent in males. Relatedly, Kendler and Myers (2014) report sex-specific patterns in disorders associated with the genetic likelihood for externalizing and internalizing behaviors, emphasizing the need to consider sex-specific pathways to better understand these behaviors.

Reporter source is another key moderator: heritability tends to be higher for other-reported data than for self-reports, likely reflecting discrepancies in perception (Nikstat and Riemann, 2020). Externalizing behaviors generally show higher heritability than internalizing behaviors (Nikstat and Riemann, 2020), though this may partly reflect greater difficulty of measuring internalizing behaviors, which involve processes within the self. Nonetheless, both domains exhibit overlapping as well as unique genetic liabilities (Hicks et al., 2004; Franić et al., 2014; Kendler et al., 2016).

GWASs on socioemotional behaviors have begun to illuminate their molecular underpinnings. Karlsson Linnér et al. (2021) identify over 500 genome-wide significant SNPs associated with traits related to self-regulation and addiction in a GWAS of 1.5 million individuals. Polygenic indices (PGIs), aggregating over SNP (genetic loci) associations estimated from these GWASs predict a range of behavioral and medical outcomes, including opioid use disorder, suicide, and unemployment.

However, findings for ASEBA-relevant domains remain limited. A family-based GWAS by Barr et al. (2020) found no robust genome-wide associations for externalizing behaviors, likely due to limited sample size (N=7,568). Similarly, a meta-analysis of univariate GWASs on internalizing symptoms by Jami et al. (2022) reported low SNP heritability (~2%) and no genome-wide significant hits in a meta-analysis of internalizing symptoms—again likely a result of limited sample size (N=64,561)— although self-reported symptoms showed somewhat higher heritability (6%) than other-reported ones—again underscoring the importance of reporter heterogeneity.

Building on these insights, this study aims to address the following research questions:

RQ1 To what extent are educational attainment (EA)-related genetics robustly associated with socioemotional behaviors?
RQ2 To what extent do these robust associations run through cognitive (Cog) and non-cognitive (NonCog) educational attainment (EA)-related genetics?
RQ3 To what extent do these robust associations differ between boys and girls or reporter source (parental and self-reported outcomes)?

By identifying robust genetic associations, distinguishing between cognitive and non-cognitive genetic pathways, and by comparing parental and offspring self-reported measures, this study provides nuanced insight into the genetic and environmental influences on socio-emotional development.

This study leverages the Lifelines genotyped (and partially imputed) trio sample to investigate these questions. We employ PGIs for educational attainment in a within-family design, controlling for parental genetics. Incorporating parental genotypes allows us to distinguish direct genetic effects in the offspring from the total genetic effects, which are biased by indirect genetic effects, population stratification and non-random mating. We define the direct effect as the association between offspring genetics and offspring outcomes after controlling for parental genetics^2^.

Furthermore, this study design allows for a causal interpretation of direct genetic effects. This is because Mendelian inheritance gives rise to a natural experiment whereby conditional on parental genotype the offspring’s genotype is as good as random. Such a conceptualization of ”causality” relies on counterfactual reasoning (Biroli et al., 2025; Harden, 2021), which considers what would have happened if an individual had inherited a different genetic variant from their parents while holding environmental factors constant. A direct test of such counterfactuals is unfeasible—arguably also unethical—in practice, which is why within-family and trio-based designs prove useful (see section PGI construction for a detailed explanation of PGI derivation and how they are used to support causal inference)

It is necessary to emphasize that the identification of causal genetic effects does not imply biological determinism; rather, genetic influences unfold through complex and long causal chains involving gene-environment interactions. The same inherited genetic variant may lead to different behavioral outcomes depending on external conditions. This perspective is particularly relevant when interpreting PGIs, which aggregate the estimated additive effects of millions of SNPs each with small effects. Although social scientists often describe PGIs as proxies for a “genetic propensity” or “genetic endowment” for a trait, such terms can be misleading, as they may suggest that genetic effects are fixed or innate. PGIs are better understood as statistical summaries of genetic associations that are shaped by the environmental and social structures in which genetic differences are expressed. Even when using within-family designs, PGIs still correlate with environmental selection processes—such as individuals actively choosing certain environments (active rGE) or eliciting particular responses from others (evocative rGE)—even though they do not directly estimate these mechanisms. In this sense, PGIs can be conceptualized as an aggregate of active and evocative gene-by-environment interactions (G×E) (Biroli et al., 2025). It is therefore crucial to interpret their effects within a broader biopsychosocial framework, reflecting how genetic influences unfold dynamically through environmental contingencies.

## 2 Data

### 2.1 Source

Lifelines is a multi-disciplinary prospective population-based cohort study examining in a unique three-generation design the health and health-related behaviours of 167,729 persons living in the North of the Netherlands^3^. It employs a broad range of investigative procedures in assessing the biomedical, socio-demographic, behavioural, physical and psychological factors which contribute to the health and disease of the general population, with a special focus on multi-morbidity and complex genetics. Lifelines data has been collected in three waves (2007–2013; 2014–2017; 2019–2023).

### 2.2 Genetic data processing

Lifelines comprises two major genotyped cohorts: CytoSNP and UGLI. The CytoSNP cohort includes only unrelated adults aged 18 and over, and is therefore excluded from our analysis. Instead, we utilize the UGLI cohort, which consists of 64,589 individuals aged eight years and older, genotyped using the Infinium Global Screening Array (691,072 SNPs). A series of rigorous quality control (QC) procedures (Marees et al., 2018) were applied to the genetic data obtained from the Lifelines Biobank. Unreliable genetic information was removed, such as multiallelic SNPs, low-frequency loci (minor allele frequency <1%), and SNPs with low imputation quality (info score <0.8). Additionally, participants with excess homozygosity were excluded, as were SNPs that violated Hardy-Weinberg Equilibrium at a stringent threshold (*p* < 10^−6^).

Since many participants were missing genetic data, we imputed parental genotypes using Mendelian imputation (Young et al., 2022). This approach infers the parental genotype by leveraging data from a genotyped sibling or a single genotyped parent. Mendelian imputation relies on the principle that siblings inherit different combinations of their parents’ genetic material, allowing for a statistical reconstruction of the unobserved parental genome. This technique provides an unbiased estimate of parental genetic effects while minimizing measurement error (Young et al., 2022).

### 2.3 Final sample

We focus on children aged 6–18 years with genotyped data available for both offspring and parents (i.e., genetic trios, with some parental genotypes imputed). This final analysis sample consists of 3,960 unique individuals, contributing a total of 7,692 observations, of which 6,219 include at least one imputed parental genotype. A majority of individuals (81.3%) are observed more than once, and 12.9% are observed more than twice. The observations are drawn from two versions of the Achenbach System of Empirically Based Assessment (ASEBA): 4,554 from the self-reported Youth Self-Report (YSR) and 3,138 from the parent-reported Child Behavior Checklist (CBCL). For more details, see subsection Problem behavior as assessed by ASEBA.

### 2.4 PGI construction

After quality control, PGIs for EA were constructed. PGIs quantify genetic influences on complex traits by summarizing associations between ~ million genetic independent loci and a particular phenotype (Becker et al., 2021). They are particularly useful for polygenic traits like educational attainment, where millions of SNPs each have small associations with the outcome (Mills et al., 2020). Essentially, a PGI is a weighted sum of the tiny associations of these individual’s SNPs, capturing their combined influence on a given trait:

$$PGI_{i}=\left(\begin{array}{lll} x_{i,1} & \ldots & x_{i,J} \end{array}\right)\left(\begin{array}{c} w_{1}\\ \vdots \\ w_{J} \end{array}\right)$$

Here, $x_{i,j}$ represents the allele count at SNP j for individual i, combined with its weight $w_{j}$, derived from genome-wide association studies (GWAS). In GWAS, each SNP is regressed on years of schooling, with controls for sex, age, and genetic principal components to minimize spurious associations due to ancestral differences. These regression coefficients serve as the SNP-weights in the PGI, enhancing prediction for polygenic traits like educational attainment.

PGIs were constructed leveraging the latest genome-wide association study (GWAS). More specifically, we used two sets of polygenic indices as predictors. The first includes the full educational attainment (EA) PGI, derived from the EA GWAS conducted by Okbay et al. (2022) (excluding the 23andMe sample), which explains 12–16% of the variation in educational attainment between individuals. The second includes educational attainment PGIs decomposed into cognitive (Cog) and non-cognitive (NonCog) components, obtained using GWAS-by-subtraction (Demange et al., 2021) with weights provided by Lee et al. (2018)^4^.

Given that standard GWAS-based PGI estimates can be biased due to sample overlap and population structure, MetaSubtract was applied to correct for potential biases (Nolte, 2020). In other words, we removed Lifelines-related contributions to the GWAS from the discovery GWAS dataset. To maximize predictive power, SBayesR, a Bayesian regression approach that accounts for linkage disequilibrium, was used to ensure that SNP weights were optimally adjusted Lloyd-Jones et al. 2019. The resulting PGI was standardized to allow for comparability between individuals.

In line with the aims of our study, we construct PGIs not only for the offspring but also one for the parents by summing the PGIs of both individual parents. This enables us to leverage triobased designs and Mendelian inheritance and ensures that, conditional on parental genotypes, the child’s genotype is as good as randomly assigned (see Introduction for a longer discussion) This principle underpins our identification strategy and justifies causal interpretation. Visual evidence supporting the randomness of genetic transmission is provided in Figure 1 of the Appendix

### 2.5 Problem behavior as assessed by ASEBA

The Lifelines study uses modified versions of the Child Behavior Checklist (CBCL) and Youth Self Report (YSR) from the Achenbach System of Empirically Based Assessment (ASEBA) (Achenbach, 2001). We use the CBCL and YSR problem behavior measures observed in by parents, as well as children and adolescents themselves. The CBCL and YSR are widely used in clinical and educational settings to identify developmental problems, monitor changes over time, and guide intervention strategies.

The CBCL6/18 is administered to the parents of children aged 8–17 years in the first wave and aged 4–12 years in subsequent waves (parental reports). The YSR is completed directly by adolescents aged 13–17 years (self reports). This age range is particularly relevant for studying how genetic influences on socioemotional behaviors evolve, as externalizing behaviors tend to stabilize in adolescence, while the heritability of internalizing behaviors increases over time (Nikstat and Riemann, 2020). Furthermore, previous findings suggest that the predictive power of PGIs varies between stages of development, reflecting differential and changing gene-environment interactions and the influence of genetic nurturing (de Zeeuw et al., 2020; Malanchini et al., 2024).

The CBL and YSR have identical response options (0: Not at all; 1: A bit or sometimes; 2: Clearly or often). Each assessment includes approximately 90 items, which are not mapped one-to-one with individual behaviors, but rather contribute to a set of empirically derived scales that reflect patterns of related symptoms. These scales are constructed by summing specific items associated with particular behavioral domains. Some of these scales are further grouped into broader categories of internalizing and externalizing problem behaviors (Achenbach, 2001).

The problem behavior measures used in this study span internalizing and externalizing domains and are displayed below from most internalizing to most externalizing. Note that three intermediate domains—Social, Thought, and Attention Problems—are placed between these groupings.

These behaviors are relevant for children’s everyday functioning, particularly in how they interact with others, engage with learning tasks, and adapt to structured environments such as schools. Internalizing behaviors may influence emotional well-being and lead to social withdrawal. The intermediate domains often reflect difficulties with attention, social engagement, or atypical patterns of thinking. Externalizing behaviors, on the other hand, may involve outward expressions of conflict or rule-breaking that challenge social expectations.

#### Internalizing Problem Behaviors:

Reflect issues directed inwardly. These are assessed through:

- **Anxious Scale**, which assesses symptoms of anxiety and depression.
- **Withdrawn Scale**, which captures symptoms of withdrawal and depression.
- **Somatic Complaints Scale**, which evaluates physical symptoms associated with emotional distress.

#### Social Problems Scale

Measures difficulties in social interactions, such as issues with forming and maintaining friendships, dependence, or isolation.

#### Thought Problems Scale:

Assesses unusual or obsessive thoughts, behaviors, and experiences, including hallucinations, compulsive actions, or bizarre ideas.

#### Attention Problems Scale:

Measures symptoms such as difficulty in concentrating, impulsivity, and hyperactivity, which are common in conditions like attention-deficit/hyperactivity disorder (ADHD).

#### Externalizing Problem Behaviors:

Outwardly directed behaviors that can disrupt social environments. These are assessed through:

- **Rule-Breaking Behavior Scale**, which includes behaviors that violate social norms and rules.
- **Aggressive Behavior Scale**, which captures conduct-related issues.

Scores are standardized within samples (CBCL and YSR samples, respectively) to facilitate comparisons across different age groups, accounting for developmental variations. The densities of these standardized scores (Figures 2–4)^5^ are presented for each problem behavior scale in the Appendix.

### 2.6 Covariates

The analyses control for several covariates (*Z*), each selected for its relevance to educational and genetic studies:

- **Offspring sex**: Binary variable to account for sex-based differences in problem behavior. Mean-centered within CBCL and YSR samples, respectively.
- **Offspring age at inclusion**: Measured in years to control for age-related variability in problem behavior. Standardized within CBCL and YSR samples, respectively.
- **Offspring birth year**: To adjust for unobserved differences across birth cohorts. Standardized within CBCL and YSR samples, respectively.
- **First 10 principal components for genetic similarity**: To account for ancestral differences within the sample. Standardized within CBCL and YSR samples, respectively.
- **Wave number**: To control for unobserved differences across collection waves. Standardized within CBCL and YSR samples, respectively.

## 3 Statistical analysis

We fit two ordinary least squares (OLS) regression models. Each model is estimated separately for the multiple outcome variables, for the two sources (self- and parental reported outcomes), and the two sets of predictors (EA, cognitive and non-cognitive PGIs). All models are estimated with the same set of controls *Z*. These include offspring’s sex, birth year, and age at inclusion, the first ten principal components of the genetic similarity, and wave number.

Following de Zeeuw et al. (2020), we set the significance threshold (alpha level) at 0.01, rather than the conventional 0.05. Using a stricter threshold helps mitigate the risk of spurious findings, given the multiple hypothesis testing. However, considering that the behavioral outcomes are correlated, we believe that applying highly stringent corrections such as the Bonferroni method—which assumes independence—would be overly punitive and likely reduce statistical power.

Additionally, some individuals were observed across multiple waves, introducing serial correlation in the error terms. While this does not bias point estimates of regression coefficients, it can lead to underestimation of the standard errors, thereby affecting hypothesis testing.

In principle, clustering at the individual level could account for serial correlation and within-individual dependence. In our final analysis sample, over 80% of individuals are observed more than once; however, the majority of those are observed only twice, with just 13% contributing more than two observations. This limited within-individual variation reduces the stability of standard error estimates when clustering at such a fine level. In contrast, our analytic focus is on family-level variation—leveraging PGIs from both children and parents—making the family the conceptually appropriate clustering unit. Given this, we opted to cluster standard errors at the family level. This approach strikes a practical balance—addressing within-family correlation and repeated observations—while ensuring sufficient variation for valid estimation of robust standard errors.

We employ two models: Model 1 estimates the total genetic effect measured by the PGI, where subscript *O* refers to the offspring.


(1) $$Y_{O}=\beta_{\text{total}}PGI_{O}^{EA}+Z+\epsilon$$

Model 2 adds an additional parameter for the parent’s (subscript *P*) PGI and thereby decomposes the total genetic effect into a direct effect measured by $\beta_{\text{direct}}$ and an indirect effect measured by $\beta_{\text{indirect}}$. In this model, the estimated coefficient for the offspring $PGI_{O}^{EA}$ can be interpreted as the causal effect of the PGI on the outcome $Y_{O}$.


(2) $$Y_{O}=\beta_{\text{direct}}PGI_{O}^{EA}+\beta_{\text{indirect}}PGI_{P}^{EA}+Z+\epsilon$$

To test sex differences, we ran the models with a sex-PGI interaction, as well as separately for boys and girls. In the interaction term approach, all covariates (excluding sex) are also interacted (following Keller, 2014).

If variation is observed in estimated coefficients between each sample (CBCL and YSR), a Wald test is applied to test the significance of their difference.

## 4 Results

### 4.1 Effects of full and decomposed EA PGI on socioemotional traits in parental and self-reported samples

Figures 1 and 2 display the estimated coefficients for the offspring PGIs from Models 1 and 2 in the CBCL and YSR samples, respectively. The top panels of each figure show results for the full EA PGI, while the bottom panels present results for its cognitive (Cog) and non-cognitive (NonCog) components. Model 1 captures the total genetic effect, reflecting the association between offspring PGI and behavioral outcomes without adjusting for parental genotype. Model 2, by contrast, estimates the direct genetic effect by conditioning on parental PGIs, thereby isolating the effect of the offspring’s inherited genetics from potential confounding due to shared familial environment or parental genetic influences.

#### In the parental report sample (CBCL)

(Figure 1) significant and robust associations are observed for Attention Problems, Rule-Breaking Behavior, and Externalizing Problem Behavior. All associations are negative, implying that higher EA PGIs are associated with lower scores on these ASEBA problem behavior scales.

*Attention Problems* and *Rule-Breaking Behavior* exhibit robust negative associations with both the full EA PGI (top panel) and its cognitive component (bottom panel), even after accounting for parental genetics. For Attention Problems, the direct genetic effect—captured by the coefficient on the offspring PGI in Model 2—is −0.081 (*p* < 0.002) for EA and −0.288 (*p* < 0.001) for Cog EA. For Rule-Breaking Behavior, the direct genetic effect is −0.078 (*p* < 0.002) for EA and −0.103 (*p* < 0.009) for Cog EA. Interestingly, while the NonCog EA PGI is initially significant for both behaviors in Model 1 (bottom panel), these associations are not robust to the inclusion of parental genetics. No other behavioral scale shows a significant relationship with the non-cognitive EA PGI in either model.

*Externalizing Problem Behavior* shows a statistically significant negative total genetic effect of the EA PGI. However, these effects are not robust to controlling for parental PGIs. In contrast, the Cog EA PGI demonstrates both significant total and direct effects, with a direct genetic effect of Cog EA PGI is −0.305 (*p* < 0.007).

#### In the self-reported sample (YSR)

(Figure 2) only Somatic Complaints show robust negative associations with the EA and the Cog EA PGI, including after controlling for parental genetics. The direct genetic effect is −0.115 (*p* < 0.001) for EA and −0.254 (*p* < 0.001) for Cog EA. In the self-reported sample, there were no significant NonCog EA PGI associations.

We tested for the statistical significance of the differences in the estimated EA PGI coefficients in the parental versus self-reported samples using a Wald test. At a significance level of 1%, results are statistically identical across CBCL and YSR for all scales.

Complete results for all behavioral measures are presented in Tables 1 and 2 in the Supplementary material. These tables also display the estimated coefficients for parental genetic factors, which are statistically insignificant for all observed ASEBA behavioral scales.

### 4.2 Sex Differences

**Figure 5:**
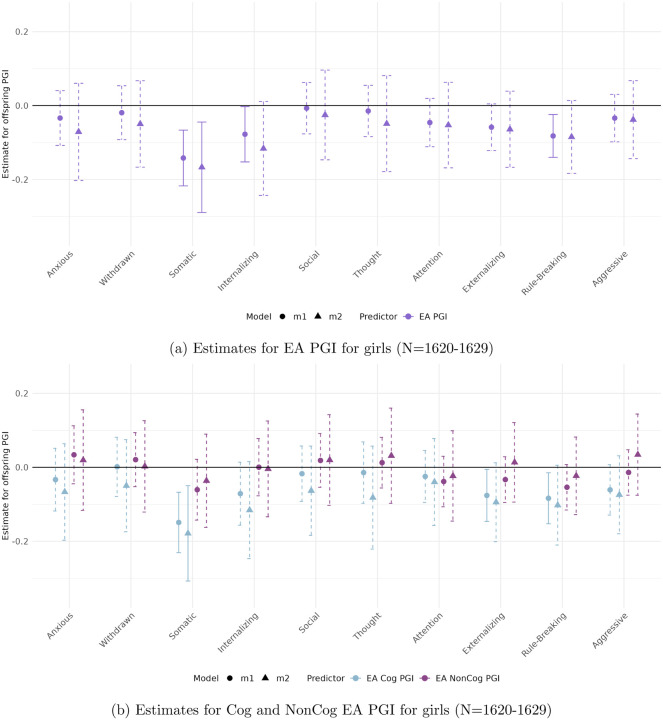
Estimated total (circles) and direct (triangles) effects of the offspring PGI on standardized ASEBA scale scores for girls in the self-reported sample (YSR). Straight lines reflect effect estimates that are significant at a stringent 0.01 significance level, while dotted lines reflect insignicant estimates.

**Figure 6:**
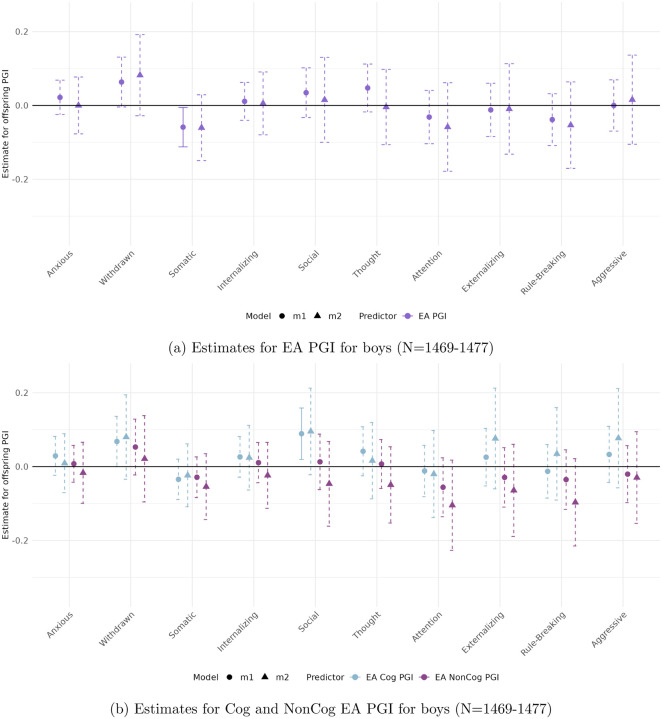
Estimated total (circles) and direct (triangles) effects of the offspring PGI on standardized ASEBA scale scores for boys in the self-reported sample (YSR). Straight lines reflect effect estimates that are significant at a stringent 0.01 significance level, while dotted lines reflect insignicant estimates.

Figures 3 to 6 present the estimated coefficients for the full and decomposed EA PGIs of the offspring from Models 1 (total effect) and 2 (direct effect), disaggregated by sex, in the CBCL and YSR samples, respectively.

#### In the parental report sample (CBCL)

(Figures 3 and 4), stratifying by sex reveals patterns consistent with the full sample, with robust associations observed for Attention Problems, Rule-Breaking Behavior, and Externalizing Problem Behavior.

Among boys, only the association between the full EA PGI and Attention Problems reaches significance (*β* = −0.105, *p* < 0.01). For girls, significant associations are observed across all three behavioral measures. These remain robust to adjustment for parental genetics only in the case of the Cog EA PGI, with direct effects of −0.319 (*p* < 0.004) for Attention Problems, −0.148 (*p* < 0.008) for Rule-Breaking Behavior, and −0.424 (*p* < 0.005) for Externalizing Problem Behavior.

Among girls, interesting patterns emerge for the Thought Problems outcome. The NonCog EA PGI has a significant coefficient *only* after controlling for parental genetics (*β* = 0.149 *p* < 0.01. Furthermore, for this outcome we observe the only statistically significant parental PGI effect across all models, the NonCog EA PGI (*β* = −0.096 *p* < 0.01, see Table 4 in the Supplementary material). Notably, the offspring and parental PGIs genetic effects exhibiting opposing signs—an unusual pattern that warrants further interpretation.

#### In the self-reported sample (YSR)

(Figures 5 and 6), sex differences are more pronounced and associations reach higher levels of statistical significance. No robust genetic associations are found for boys. In contrast, girls show a strong and consistent relationship between Somatic Complaints and both the full EA PGI (*β* = −0.167, *p* < 0.001) and the EA Cog PGI (*β* = −0.446, *p* < 0.001).

#### To formally test sex differences

we estimated interaction models including sex-PGI terms^6^. Sex interactions with the full and decomposed EA PGIs show almost no significant hits in the parental report sample, with the effect of the interaction of sex and the NonCog EA PGI on Thought Problems being the only exception (*p*_*M*1_ < 0.05 and *p*_*M*2_ < 0.009). In the self-reported sample, genetic effects for many measures show significant sex differences, especially for the Cog EA PGI. These include Attention Problems, Anxious, Somatic Complaints, Withdrawn, Social Problems, Externalizing and Internalizing Problem Behavior. From these, *Somatic Complaints* is the only measure that exhibits some significant differences for both the full and the cog EA PGI (*p*_*M*1_ < 0.02*, p*_*M*2_ < 0.08, and *p*_*M*1_ < 0.003*, p*_*M*2_ < 0.02, respectively).

Complete results by sex and sex-interactions for all behavioral measures are presented in Tables 3 to 8 in the Supplementary material. Parental genetics as a predictor remains statistically insignificant for all observed behavioral measures.

Results for Wald tests are presented in Table 9 in the Supplementary material.

## 5 Discussion

### 5.1 Main Findings

This study investigated the genetic underpinnings of (problematic) socioemotional behaviors in children and adolescents (ages 6–18) using polygenic indices (PGIs) for educational attainment (EA) and its cognitive (Cog) and non-cognitive (NonCog) components. Leveraging a trio-based, within-family design in the Lifelines cohort, we account for confounding due to population stratification, assortative mating, and indirect genetic effects, allowing for a causal interpretation of offspring PGI estimates. While not implying genetic determinism, this approach strengthens the case for genetic influence on socio-emotional traits beyond purely correlational inference. We examined whether EA-related PGIs predict socioemotional behaviors, whether cognitive and non-cognitive components show differential effects, and whether associations vary by sex or reporter source (parental vs. self-report). We find robust evidence for all three, offering novel insights into the distinct pathways through which genetics contribute to socio-emotional development.

Our findings provide four key insights: (i) EA-related genetic factors contribute positively to the development of healthier socioemotional behaviors, with cognitive genetic components predominantly driving these associations; (ii) Sex differences emerge in the genetic influences on socioemotional behaviors, with stronger genetic effects observed for females particularly for Somatic Complaints; (iii) We observe modest differences between self-reported and parental-reported measures, albeit not statistically significant; (iv) We find little robust evidence for indirect genetic effects, though some attenuations after controlling for parental genetics suggest they may exist in modest form, primarily through non-cognitive pathways.

#### Cognitive vs. Non-Cognitive Genetics

We find that EA-related genetic influences on socioemotional behaviors are predominantly driven by cognitive genetic components. Behavioral measures such as Attention Problems and Rule-Breaking Behavior exhibit robust associations with both the full EA PGI and the cognitive EA PGI, whereas non-cognitive genetic influences are weaker or absent. These results align with prior studies that emphasize the importance of cognitive traits in behavioral and educational outcomes (Demange et al., 2021; de Zeeuw et al., 2020).

The significant role of cognitive genetics raises critical questions. One interpretation is that problematic socioemotional behaviors are inherently linked to cognitive functioning, reflecting behavioral characteristics that align with success in standardized tests. Traits such as focus, self-regulation, and impulse control, which are essential for educational success, may also shape social behavior, explaining the strong cognitive genetic associations we observe. The developmental literature further supports a more integrated view of cognitive and behavioral traits. Best and Miller (2010) emphasize that executive functions—defined as cognitive processes orchestrated by the prefrontal cortex, including inhibition, working memory, and shifting—develop across childhood and are foundational for goal-directed behavior. While these processes are traditionally classified as “cognitive,” their role in behavior regulation highlights the limitations of sharply separating cognitive and non-cognitive domains. Rather than reflecting distinct genetic influences, the traits captured by Cog and NonCog PGIs may overlap substantially in both biological basis and developmental function. Executive function, as described by Best and Miller, may thus serve as a useful conceptual bridge for understanding how cognitive development underpins both academic and socio-emotional outcomes, even if their paper does not explicitly frame EF in those terms.

Alternatively, these findings might reflect methodological nuances in how NonCog PGIs are constructed. Although Demange et al. (2021) suggest that their methodology effectively isolates non-cognitive components, residual overlap may still influence observed associations. The GWAS-by-subtraction procedure assumes by construction that cognitive and non-cognitive genetic influences are orthogonal. This strong assumption excludes any genetic loci that contribute to both cognitive and non-cognitive traits—such as self-regulation or motivation that are partly grounded in cognitive function—from the NonCog PGI. Instead, such pleiotropic effects are captured entirely within the Cog PGI. As a result, the NonCog PGI likely captures only a subset of the genetic variance in non-cognitive contributors to educational attainment—specifically, those that are genetically uncorrelated with cognition. This modeling decision, while useful for isolating distinct components, may lead to an underestimation of the true role of non-cognitive traits in predicting behavioral outcomes. In turn, it may help explain why we observe stronger associations between socioemotional behaviors and the Cog EA PGI across our analyses.

#### Sex Differences

Our findings also reveal significant sex differences in the genetic influences on socioemotional behaviors. Girls exhibit stronger genetic associations across multiple behavioral traits, while boys show more limited associations, primarily for Attention Problems in parental-reported data.

These results align with the findings of Kendler and Myers (2014), who demonstrated that the genetic structure of internalizing and externalizing traits differs by sex. Specifically, genetic influences on internalizing traits (e.g., anxiety, depression) are stronger in females, whereas shared environmental influences play a larger role in male externalizing behaviors (Kendler et al., 2016).

A possible explanation for this is that boys’ socio-emotional development is more strongly shaped by environmental influences, such as peer interactions and family dynamics, rather than direct genetic predispositions. This aligns with prior evidence that shared environmental effects contribute more substantially to externalizing traits in males (Kendler et al., 2016). Moreover, the fact that sex-stratified GWASs do not find substantial genetic differences (Jami et al., 2022; Okbay et al., 2022) suggests that sex-specific gene-environment interactions may drive the observed disparities.

#### Reporter Variability

Although prior research has highlighted substantial differences in genetic associations depending on the source of behavioral reports—particularly for internalizing symptoms (Jami et al., 2022)—our findings suggest only modest and statistically insignificant variation between self-reported and parental-reported outcomes. For example, while self-reported Somatic Complaints showed robust associations with EA and Cog EA PGIs, and parental-reported Attention Problems and Rule-Breaking Behavior were more strongly associated with cognitive genetics, these patterns did not reach statistical significance when directly compared across reporter types. This may reflect true similarities in underlying genetic influences across sources, or limitations in statistical power. Nonetheless, our results are directionally consistent with the notion that self-reports may better capture internalized emotional states, whereas parental reports may more reliably reflect observable, externalizing behaviors. As such, even in the absence of strong statistical differences, the choice of reporter remains an important methodological consideration for genetic studies of behavioral traits.

#### Lack of Robust Evidence for Indirect Genetic Effects

We find little robust evidence for indirect genetic effects of parental EA-related genetics on offspring socioemotional behaviors. Parental PGIs show no statistically significant associations with any socio-emotional traits, and for most outcomes, the estimated effects of the offspring PGIs remain virtually unchanged after conditioning on parental genetics.

These findings are consistent with those of Tanksley et al. (2025), who examined the genetic underpinnings of child externalizing behavior using an externalizing PGI derived from a large-scale adult GWAS of self-regulatory traits (e.g., ADHD, problematic alcohol use, cannabis use, risk tolerance). Together, these results could suggest that indirect genetic effects—or gene-environment correlations—may play a more limited role in shaping socio-emotional behaviors than they do in shaping educational outcomes.

They are also in line with prior findings from Dutch cohorts, such as Demange et al. (2022), who reported similarly limited evidence for indirect genetic effects on educational outcomes. These parallels may reflect contextual factors specific to the Netherlands, such as its relatively egalitarian social structure and parenting norms that emphasize autonomy and peer influence. Our final analysis sample (3,960 individuals from 2,469 families) is comparable in size—and in fact slightly larger—than the trio sample used by Demange et al. (2022), suggesting that our null findings are unlikely to be driven by a smaller or less informative design.

An exception to this pattern emerged in the parental reported Thought Problems outcome for girls, where we observed the only statistically significant parental PGI effect across the full set of models. Notably, this association is accompanied by direct and indirect genetic effects with opposing signs—an unusual pattern that may indicate an environmentally mediated influence of parental EA-related traits that contrasts with the child’s own genetic likelihood. One possible interpretation is that parents with high EA-related PGIs may shape environments marked by high cognitive demand, structure, or achievement expectations, which could heighten internalizing or obsessive tendencies in daughters. Alternatively, this may reflect reporter bias, whereby higher-PGI parents are more attentive to or more likely to report subtle or subclinical psychological symptoms.

Some attenuation in offspring PGI associations after controlling for parental genetics also warrant a more nuanced interpretation. Specifically, total genetic effects of the NonCog EA PGI on Attention Problems and Rule-Breaking Behavior, as well as the total effect of the full EA PGI on Externalizing Problem Behavior, became non-significant once parental genetics were included in the model. In sex-stratified analyses, similar attenuation patterns emerged among girls, where associations of the full EA PGI with Attention Problems, Rule-Breaking Behavior, and Externalizing Problem Behavior lost significance after adjusting for parental PGIs. These patterns could reflect weak environmentally mediated parental influences—such as parental behaviors correlated with their own genetic predispositions shaping the child’s social environment—consistent with modest indirect genetic effects or gene–environment correlation processes (e.g., active or evocative rGE).

At the same time, the overall pattern of results suggests that any indirect effects are likely to be small. While our simulation-based power analysis demonstrates high sensitivity to modest genetic effects (e.g., *β* ≥ 0.10, see Appendix), power for detecting smaller effects is substantially lower. Given that indirect genetic effects are typically weaker, our models may lack sufficient power to reliably detect them. This limitation is compounded by potential measurement constraints: the behavioral measures used here are based on parental and self-reports rather than teacher reports, which have been shown to more reliably capture socioemotional behaviors (Feng et al., 2022). The absence of such third-party reports may further reduce sensitivity to environmentally mediated parental influences.

Future research with larger samples, cross-national comparisons, and richer environmental measures (e.g., teacher or school-based assessments) will be crucial to determine whether these attenuations reflect genuine indirect genetic effects or statistical noise. Notably, the direct genetic effects of the Cog EA PGI remain robust across nearly all traits—even in models adjusting for parental genetics—suggesting that the primary causal pathway linking EA-related genetics to socioemotional behaviors operates through cognitive rather than non-cognitive mechanisms. To the extent that indirect effects exist, they appear more likely to operate through non-cognitive pathways, which are weaker and less consistently associated with problem behaviors in this cohort.

### 5.2 Implications

These findings offer several important implications for research, policy, and intervention. First, the robust associations between cognitive genetic factors and problematic socioemotional behaviors highlight the potential of cognitive-based interventions—such as those targeting executive functioning, attentional control, and self-regulation—not only to support academic achievement but also to improve behavioral outcomes. This reinforces our understanding about the interconnected nature of cognitive and socio-emotional development.

Second, although reporter differences in genetic associations were modest and statistically insignificant, the distinct patterns observed for internalizing versus externalizing traits suggest that multi-informant approaches remain valuable. Self-reports may be more sensitive to internal emotional states, while parental reports more accurately reflect external behaviors. Future assessments and interventions should consider the complementary insights offered by each perspective.

Third, the observed sex differences in genetic effects—particularly the stronger associations found among girls for internalizing symptoms—point to the need for sex-sensitive intervention strategies. Tailoring supports to the differential genetic and environmental influences across sexes may improve the effectiveness of socio-emotional development programs.

Lastly, while we find little robust evidence for indirect genetic effects, some of our results attenuations suggest that environmentally mediated effects may exist, particularly through non-cognitive pathways. In more egalitarian societies like the Netherlands, with parenting norms that emphasize autonomy and peer influence, such effects may be attenuated, highlighting the need for cross-national and school-based studies to examine how social and cultural contexts shape the strength of gene–environment correlations.

### 5.3 Limitations and Future Directions

Despite its strengths, this study has several important limitations. First, although PGIs offer a powerful tool for estimating genetic influences, they currently explain only a modest proportion of the variance in socio-emotional outcomes. The EA-related PGIs used here—particularly the non-cognitive component—may not fully capture the relevant genetic architecture of socioemotional behaviors. Continued refinement of GWAS discovery efforts may help to improve predictive power.

Second, the behavioral phenotypes analyzed were derived from standardized ASEBA instruments (CBCL and YSR), which, while validated and widely used, may not encompass the full spectrum of socio-emotional behaviors. These instruments may miss subtle, situational, or culturally specific behaviors, and rely on self- or parent-reporting, which can introduce measurement error and informant biases. The absence of teacher-reported or observational data may limit the detection of environmentally mediated or context-dependent behaviors. Future work could benefit from integrating broader behavioral assessments.

Third, while we observe sex differences in genetic associations, the underlying mechanisms remain unclear. It is uncertain whether these differences reflect developmental trajectories, environmental buffering, or sex-specific gene–environment interactions. Addressing these questions will require more targeted examination of moderating factors and longitudinal developmental processes.

Fourth, our sample size—though substantial for trio-based analyses—remains modest relative to the statistical power typically required in polygenic research. Simulation-based power analyses (see Appendix) indicate that our design is sufficiently powered (≥ 99.8%) to detect modest genetic effects (*β* ≥ 0.10), even at the lower end of our sample range (N = 3,090). However, statistical power is meaningfully reduced for smaller effect sizes, particularly for subgroup analyses. As such, null or inconsistent findings for non-cognitive PGIs or reporter differences may reflect limitations in statistical power rather than the absence of true underlying effects. Larger samples, ideally spanning multiple cohorts, are needed to strengthen the robustness and generalizability of these findings.

Lastly, our findings are based on a single cohort drawn from the Netherlands—a country characterized by relatively low socioeconomic inequality and parenting norms that emphasize autonomy. These contextual features may attenuate indirect genetic effects and limit the generalizability of our findings. While the within-family design controls for many confounders, broader influences—such as school environments, peer dynamics, and community context—are not directly captured. Future research should seek to replicate these findings across more diverse populations, including cohorts of non-European ancestry and different sociocultural backgrounds, to better understand the universality or specificity of genetic effects on socio-emotional development.

## Supplementary Material

Supplementary Files

This is a list of supplementary files associated with this preprint. Click to download.

- Tables.xlsx

Tables are available in the Supplementary Files section.

## Figures and Tables

**Figure 1:**
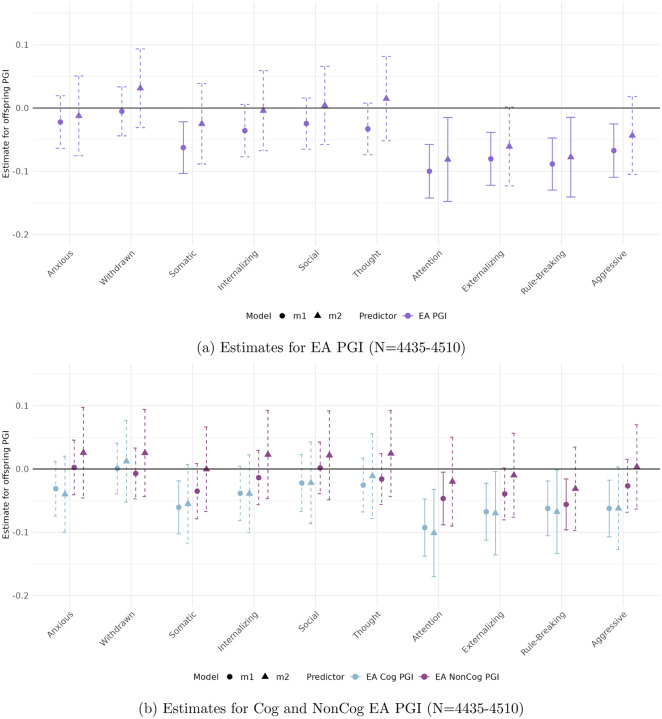
Estimated total (circles) and direct (triangles) effects of the offspring PGI on standardized ASEBA scale scores in the parental report sample (CBCL). Straight lines reflect effect estimates that are significant at a stringent 0.01 significance level, while dotted lines reflect insignificant estimates.

**Figure 2:**
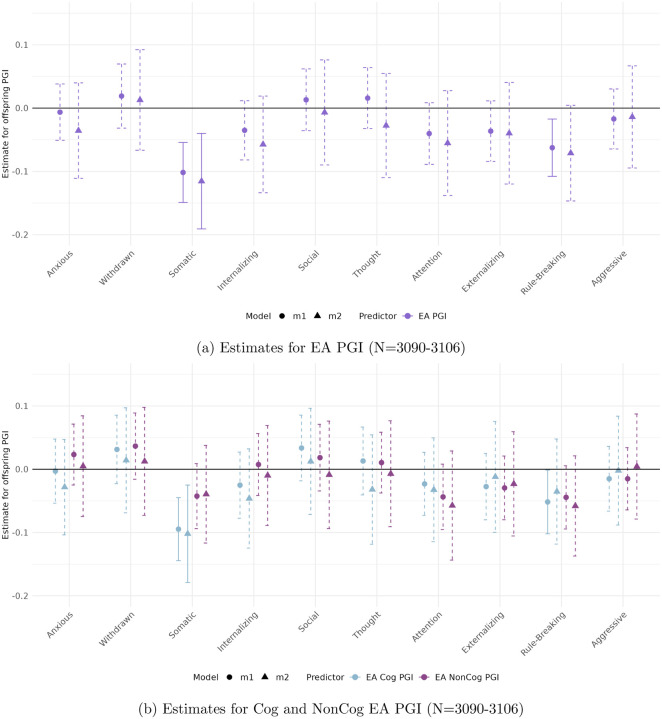
Estimated total (circles) and direct (triangles) effects of the offspring PGI on standardized ASEBA scale scores in the self-reported sample (YSR). Straight lines reflect effect estimates that are significant at a stringent 0.01 significance level, while dotted lines reflect insignicant estimates.

**Figure 3:**
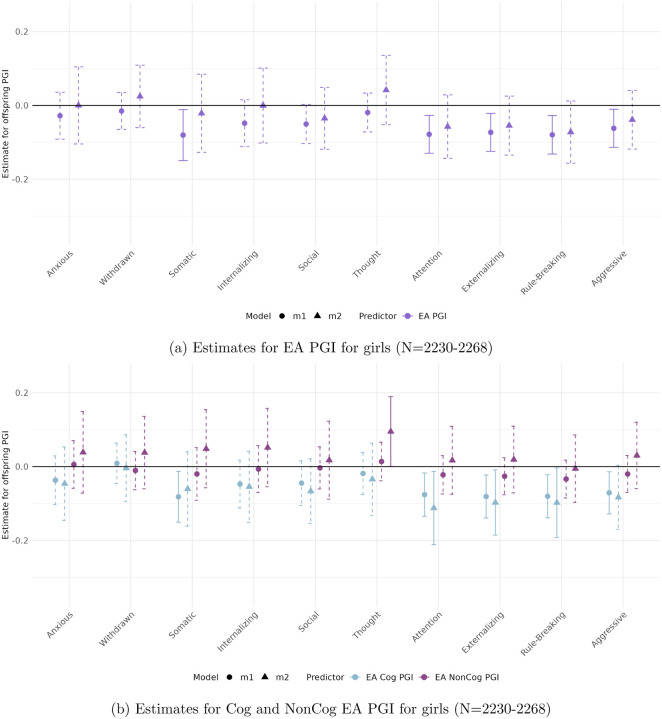
Estimated total (circles) and direct (triangles) effects of the offspring PGI on standardized ASEBA scale scores for girls in the parental report sample (CBCL). Straight lines reflect effect estimates that are significant at a stringent 0.01 significance level, while dotted lines reflect insignificant estimates.

**Figure 4:**
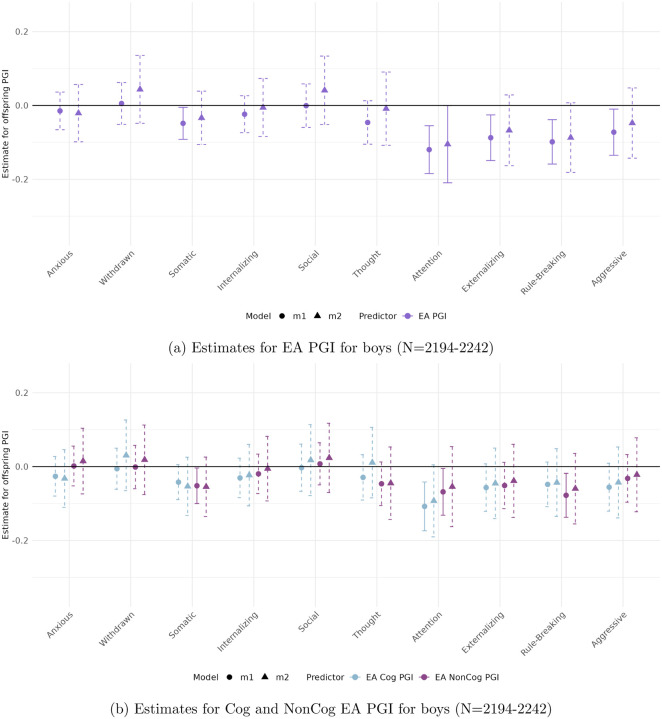
Estimated total (circles) and direct (triangles) effects of the offspring PGI on standardized ASEBA scale scores for boys in the parental report sample (CBCL). Straight lines reflect effect estimates that are significant at a stringent 0.01 significance level, while dotted lines reflect insignificant estimates.

**Figure 1:**
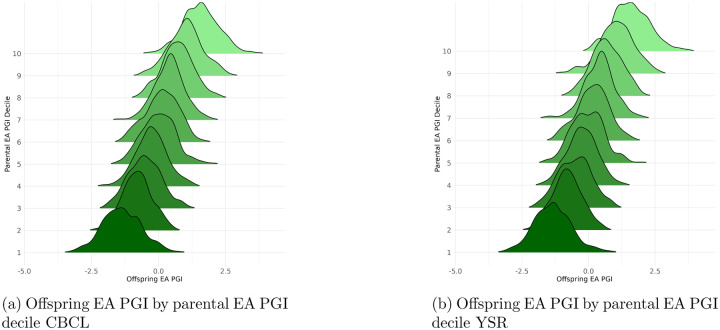
Distribution of offspring EA PGI by parental EA PGI decile in the CBCL and YSR samples

**Figure 2:**
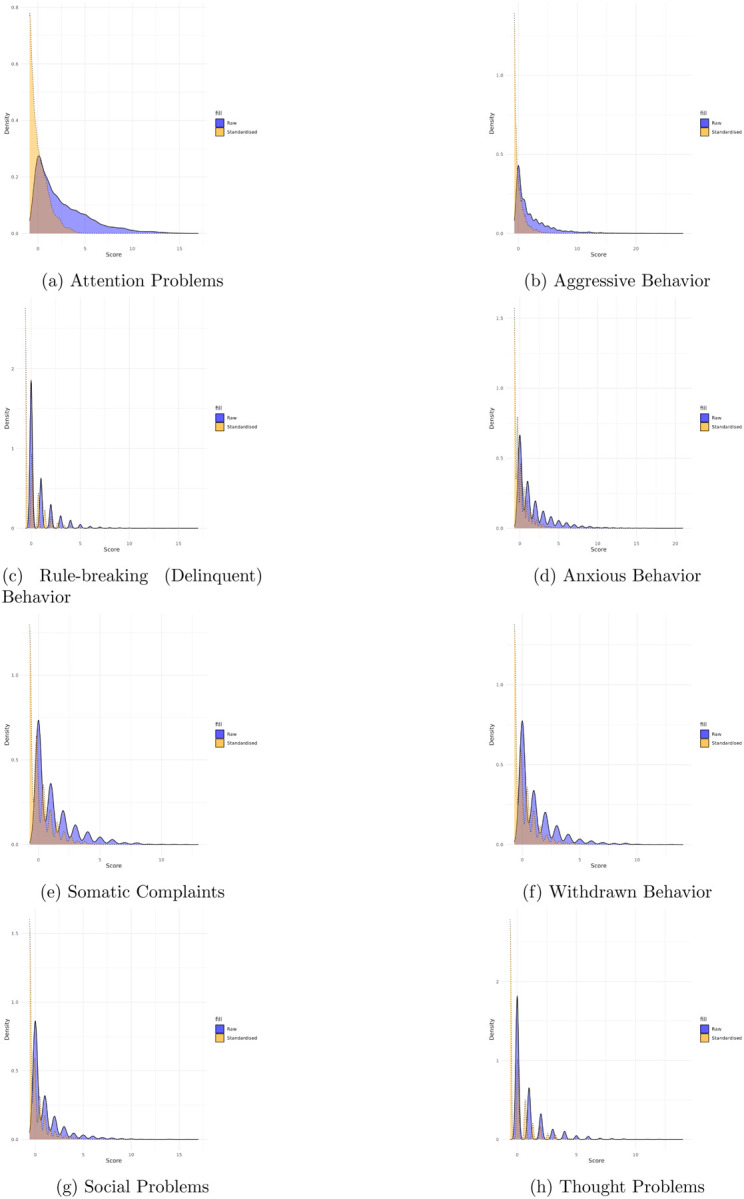
Overlaid density plots for behavioral problem measures in the CBCL sample

**Figure 3:**
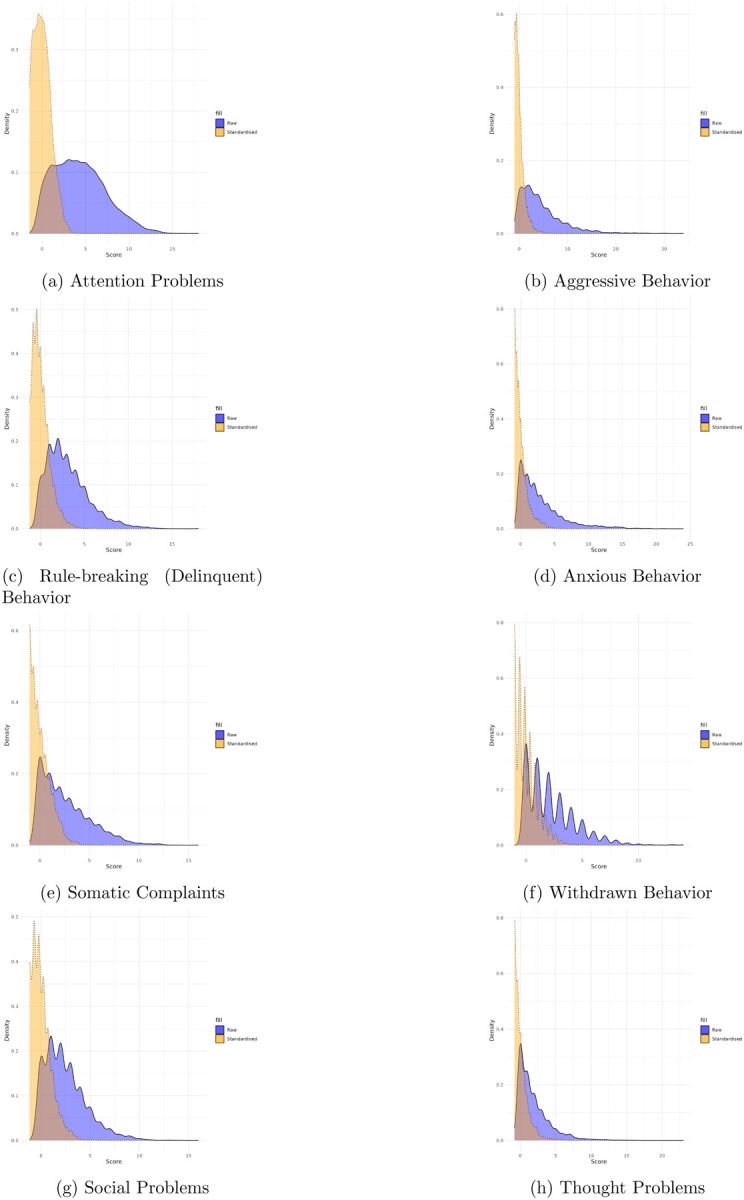
Overlaid density plots for behavioral problem measures in the YSR sample

**Figure 4:**
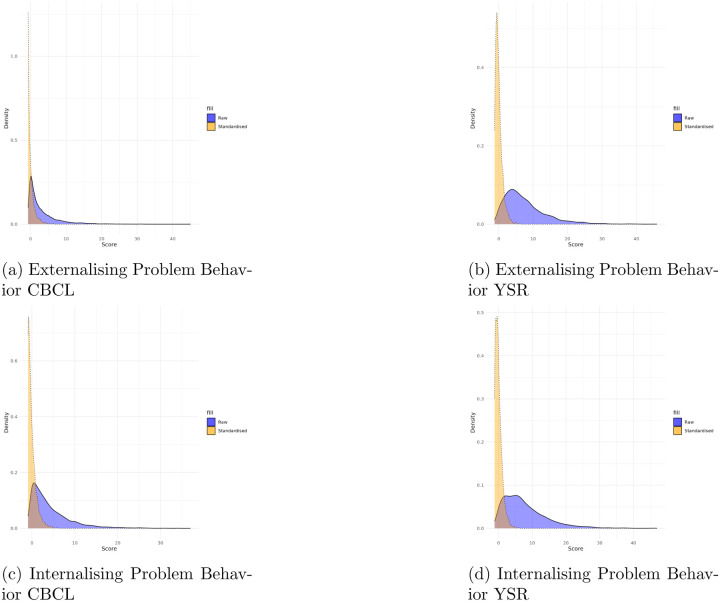
Overlaid density plots for externalising and internalising problem measures in the CBCL and YSR samples

## Footnotes

1 Recent economic literature terms this socio-emotional skills, a shift in terminology from non-cognitive skills — a misnomer that defines these attributes by what they are not (i.e., cognitive test scores) — toward a more precise conceptualization.

2 The term ”direct genetic effect” is commonly used in the literature to describe genetic associations that remain after accounting for between-family confounds. However, it remains questionable whether any genetic effect can truly be ”direct”. Even when controlling for parental genetics, children’s traits are shaped by broader environmental influences, such as peer and teacher interactions, which may themselves be correlated with genetic predispositions. In this sense, genetic effects—even those identified within-family—generally reflect indirect pathways that extend beyond parental transmission. This highlights a broader limitation in terminology: language may fail to fully capture the complexity of gene-environment interplay.

3 Drenthe, Friesland, Groningen

4 Apart from the 23andMe sample the samples used in Lee et al. (2018) and Okbay et al. (2022) are identical.

5 These are overlaid with densities of raw scores to facilitate comparison.

6 In the interaction term approach, all covariates (excluding sex) are also interacted

## Appendix

**Table 1:** Simulation-based power estimates by sample size (*N*), effect size (*β*), and observed intra-cluster correlation (ICC).

N	Clusters	*β*	Mean ICC	Power
3,090	1,931	0.05	0.083	0.556
3,090	1,931	0.10	0.083	0.998
3,090	1,931	0.15	0.080	1.000
3,090	1,931	0.20	0.080	1.000
3,090	1,931	0.25	0.079	1.000
3,090	1,931	0.30	0.078	1.000
4,510	2,469	0.05	0.082	0.758
4,510	2,469	0.10	0.082	1.000
4,510	2,469	0.15	0.080	1.000
4,510	2,469	0.20	0.081	1.000
4,510	2,469	0.25	0.078	1.000
4,510	2,469	0.30	0.076	1.000

### Simulation-Based Power Analysis

To evaluate the statistical power of our within-family trio design to detect direct genetic effects on socio-emotional outcomes, we conducted a simulation-based power analysis using Monte Carlo methods. This approach mirrors our empirical modeling framework, which includes multiple covariates and accounts for within-cluster dependence via clustered standard errors.

We simulated continuous outcomes under varying assumptions about the effect size of a polygenic index (PGI) predictor (*β*), and observed sample size (*N*), and family clustering. The key parameters were as follows:

- **Effect sizes (***β***):** 0.05, 0.10, 0.15, 0.20, 0.25, 0.30
- **Sample sizes (***N***):** 3,090 (YSR data) and 4,510 (CBCL data)
- **Clusters:** 1,931 (YSR) and 2,469 (CBCL), reflecting unique family IDs
- **Predictors:** 1 PGI and 17 covariates (reflecting Model 2 and decomposed EA PGIs as predictors, the model with the smallest degree of freedom)
- **Model:** Ordinary Least Squares (OLS) regression with clustered standard errors at the family level
- **Simulations:** 500 repetitions per condition
- **Significance threshold:** *α* = 0.01
- **ICC estimation:** In each iteration, we estimated the intra-cluster correlation (ICC) using a random-intercept model fit via lmer, defined as the proportion of variance explained by family-level clustering.

The results presented in Table 1 in this Appendix demonstrate that our design is well-powered (≥ 99.8%) to detect modest genetic effects (*β* ≥ 0.10) across both YSR and CBCL sample sizes. For smaller effects (*β* = 0.05), statistical power is more limited—56% for *N* = 3, 090 and 76% for *N* = 4,510—highlighting the sensitivity gains afforded by larger samples. Mean ICCs across conditions were approximately 0.08, indicating moderate within-family dependence and validating the use of clustered standard errors.

The results presented in Table 1 demonstrate that our design is well-powered (≥ 99.8%) to detect modest genetic effects (*β* ≥ 0.10) across both YSR and CBCL sample sizes. For smaller effects (*β* = 0.05), statistical power is more limited—56% for *N* = 3,090 and 76% for *N* = 4,510—highlighting the sensitivity gains afforded by larger samples. Mean ICCs across conditions were approximately 0.08, indicating moderate within-family dependence and validating the use of clustered standard errors.
